# Supplementary material for: Identification of a High-Risk Group of New-Onset Cardiovascular Disease in Occupational Drivers by Analyzing Heart Rate Variability
Source: Int J Environ Res Public Health. 2021 Oct 31;18(21):11486. doi: 10.3390/ijerph182111486 (PMC8582774; doi:10.3390/ijerph182111486)
Supplement: Supplementary file 1 [file ijerph-18-11486-s001.zip › ijerph-1425697-supplementary.pdf]

Table S1. Definition of HRV measures.

Parameter	Unit	Description
**Time-domain measures**		
SDNN	ms	Standard deviation of normal RR intervals
RMSSD	ms	Root mean square of successive RR interval differences
**Frequency-domain measures**		
LF power	ms^2	Absolute power of the low-frequency band (0.04–0.15 Hz)
HF power	ms^2	Absolute power of the high-frequency band (0.15–0.40 Hz)
LF/HF	%	Ratio of LF-to-HF power

RR intervals = interbeat intervals between all successive heartbeats.

Table S2. Compared HRV index for cardiovascular diagnostic categories in study population (n=788) [a. b.]

			SDNN(ms)			RMSSD(ms)			LF(ms^2)			HF (ms^2)			LF/HF		
		n	*mean*	*sd*	*p-value*	*mean*	*sd*	*p-value*	*mean*	*sd*	*p-value*	*mean*	*sd*	*p-value*	*mean*	*sd*	*p-value*
**Cardiovascular disease (ICD-9-CM: 390-459)**	No	627	30.5	15.3	0.015	19.1	13.8	0.137	341.8	499.8	0.005	149.1	349.9	0.053	3.57	3.55	0.249
	Yes	161	29.1	24.7		19.2	18.8		268.5	464.7		124.8	193.5		3.34	3.98	
Cardiovascular disease (not included Hypertensive disease) (ICD9: 391, 392.0, 393-398, 410-414, 416, 420-429)	No	739	29.9	15.0	0.433	18.8	13.3	0.922	324.7	472.1	0.203	144.0	326.4	0.081	3.48	3.47	0.650
	Yes	49	34.2	40.5		24.4	30.5		357.7	751.2		145.9	291.1		4.15	5.54	
Hypertensive disease (ICD9: 401-405)	No	660	30.8	17.1	<0.001	19.4	14.9	0.021	342.3	498.9	0.005	148.8	343.6	0.029	3.53	3.50	0.398
	Yes	128	27.1	19.9		17.5	14.9		246.9	458.2		120.3	194.9		3.46	4.31	
Ischemic heart disease (ICD9: 410-414)	No	753	30.1	16.5	0.776	18.9	14.1	0.971	324.6	474.6	0.645	143.4	325.0	0.622	3.50	3.56	0.970
	Yes	35	32.8	34.0		23.4	27.4		374.2	807.9		159.1	310.5		3.93	5.02	
Congestive heart failure (CHF) (ICD9: 398.91, 422, 425, 428, 402.x1, 404.x1, 404.x3)	No	773	30.0	16.3	0.947	18.9	14.2	0.515	326.5	492.0	0.501	143.9	325.5	0.332	3.49	3.54	0.714
	Yes	15	40.3	52.1		29.8	35.8		339.7	583.8		157.0	254.0		4.90	7.02	

a. The selection criterial for CVD events was the least five clinical visit records within a year or at least one inpatient record

b. Assess the difference in mean natural log (Ln)-transformed markers

Table S3. Hazard ratios and 95% confidence intervals for cardiovascular diagnostic categories by HRV index in the study population (n = 788).

		Cardiovascular disease (not included Hypertensive disease)				Hypertensive disease				Ischemic heart disease				Congestive heart failure (CHF)			
	Independent variables [c.]	*Hazard Ratio*	*95%CI*		*p-value*	*Hazard Ratio*	*95%CI*		*p-value*	*Hazard Ratio*	*95%CI*		*p-value*	*Hazard Ratio*	*95%CI*		*p-value*
Model 1 [a.]																	
1	As a continuous (LnSDNN)	0.73	0.40	1.35	0.32	0.57	0.39	0.83	0.001	0.99	0.48	2.06	0.98	1.14	0.38	3.41	0.82
2	As a categorical variable: SDNN (≤30 vs. >30)	2.00	1.04	3.85	0.04	1.51	1.02	2.23	0.04	1.42	0.68	2.98	0.35	1.64	0.50	5.41	0.42
3	As a continuous (LnRMSSD)	0.96	0.58	1.57	0.86	0.74	0.54	1.02	0.06	1.07	0.59	1.94	0.82	1.94	0.82	4.57	0.13
4	As a categorical variable: RMSSD (≤20 vs. >20)	1.54	0.81	2.94	0.19	1.48	0.99	2.21	0.06	1.17	0.57	2.41	0.68	0.93	0.31	2.78	0.89
5	As a continuous (LnLF)	0.87	0.68	1.11	0.26	0.83	0.71	0.97	0.02	0.99	0.74	1.34	0.97	0.97	0.60	1.55	0.88
6	As a categorical variable: LF (≤380 vs. >380)	1.36	0.65	2.86	0.41	1.28	0.82	2.01	0.28	1.11	0.48	2.58	0.81	0.95	0.25	3.55	0.94
7	As a continuous (LnHF)	0.83	0.65	1.05	0.12	0.90	0.77	1.04	0.15	0.98	0.73	1.31	0.87	0.89	0.56	1.43	0.63
8	As a categorical variable: HF (≤168 vs. >168)	1.06	0.53	2.12	0.87	1.18	0.76	1.85	0.46	0.86	0.40	1.86	0.70	0.53	0.15	1.80	0.31
9	As a continuous (LnLF/HF)	1.08	0.80	1.46	0.60	0.89	0.74	1.08	0.24	1.03	0.73	1.45	0.88	1.12	0.64	1.94	0.70
10	As a categorical variable: LF/HF (≤3.5 vs. >3.5)	0.76	0.43	1.34	0.34	1.25	0.85	1.83	0.25	0.77	0.39	1.52	0.45	0.98	0.33	2.91	0.97
Model 2 [a.]																	
11	As a continuous (LnSDNN)	0.85	0.47	1.51	0.58	0.59	0.40	0.87	0.01	1.08	0.56	2.10	0.81	1.12	0.36	3.49	0.85
12	As a categorical variable: SDNN (≤30 vs. >30)	1.81	0.93	3.53	0.08	1.53	1.02	2.29	0.04	1.27	0.60	2.70	0.53	2.13	0.59	7.75	0.25
13	As a continuous (LnRMSSD)	1.00	0.64	1.58	1.00	0.73	0.53	1.01	0.06	1.13	0.66	1.92	0.66	1.65	0.67	4.05	0.28
14	As a categorical variable: RMSSD (≤20 vs. >20)	1.44	0.75	2.76	0.28	1.54	1.02	2.33	0.04	1.07	0.51	2.22	0.87	1.01	0.30	3.38	0.99
15	As a continuous (LnLF)	0.93	0.73	1.19	0.54	0.86	0.73	1.01	0.07	1.07	0.81	1.42	0.64	0.95	0.57	1.56	0.82
16	As a categorical variable: LF (≤380 vs. >380)	1.23	0.58	2.57	0.59	1.25	0.79	1.99	0.34	1.02	0.44	2.37	0.96	1.13	0.26	4.94	0.87
17	As a continuous (LnHF)	0.88	0.70	1.11	0.29	0.89	0.76	1.04	0.13	1.01	0.77	1.32	0.93	0.83	0.51	1.35	0.46

18	As a categorical variable: HF (≤168 vs. >168)	1.01	0.50	2.03	0.97	1.29	0.82	2.05	0.27	0.83	0.38	1.82	0.65	0.61	0.16	2.41	0.48
19	As a continuous (LnLF/HF)	1.11	0.82	1.52	0.50	0.97	0.80	1.18	0.77	1.09	0.77	1.54	0.65	1.21	0.67	2.19	0.53
20	As a categorical variable: LF/HF (≤3.5 vs. >3.5)	0.67	0.37	1.21	0.18	1.05	0.71	1.54	0.82	0.70	0.35	1.40	0.31	0.73	0.24	2.27	0.59

[a.] Model 1: Adjusted for age at first employment (≥ 45 vs. < 45 years), body mass index (> 30 vs. ≤ 30), education, drinking, smoking, exercise, time since first employment (years), and shift work.

[b.] Model 2: As Model 1 and with additional adjustments for systolic blood pressure, LnCHOL, LnTG, LnHDL, and LnAC.

[c.] Each independent variable (1–20) was separately included in the models.

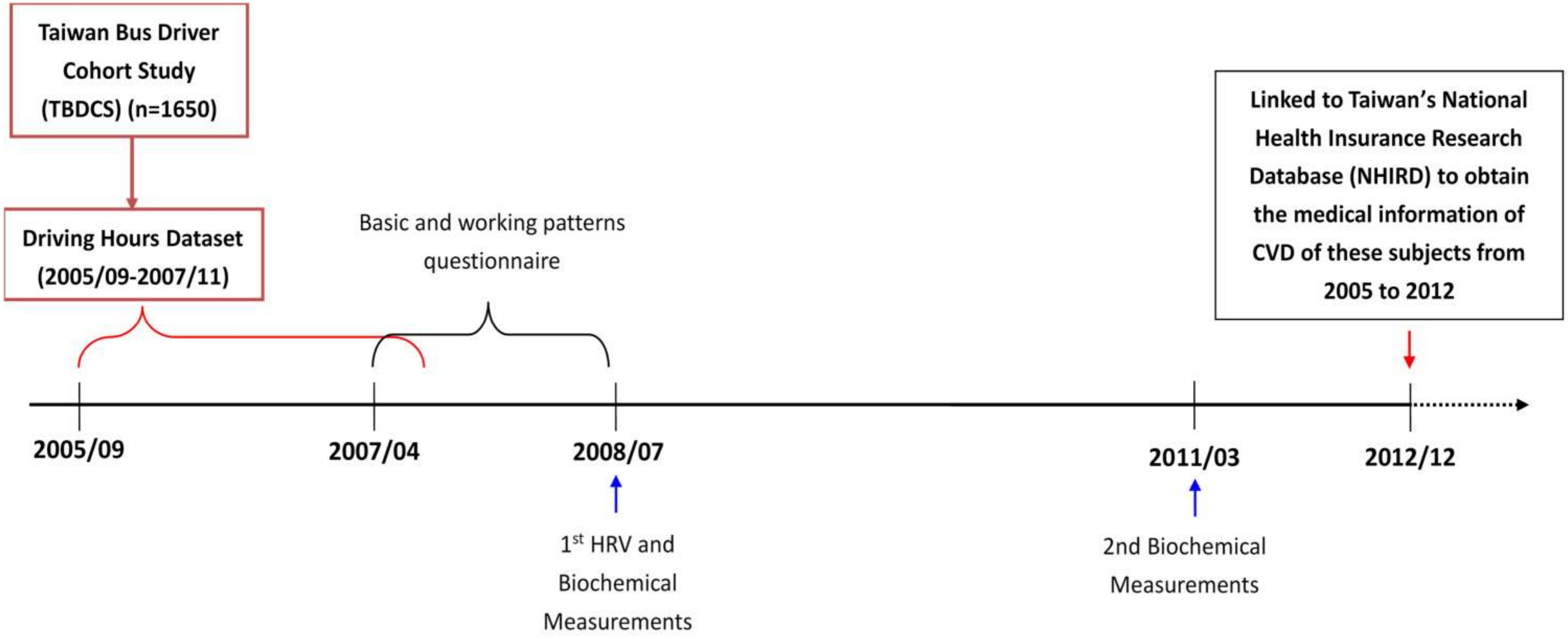

Supplement Figure S1. Taiwan Bus Driver Cohort Study (TBDCS) schedule diagram
